# Global prevalence, metabolic characteristics, and outcomes of lean-MAFLD: a systematic review and meta-analysis

**DOI:** 10.1007/s12072-025-10801-x

**Published:** 2025-03-14

**Authors:** Mark C. C. Cheah, Harry Crane, Jacob George

**Affiliations:** Storr Liver Centre, Westmead Institute for Medical Research, Westmead Hospital and University of Sydney, Westmead, New South Wales Australia

**Keywords:** Metabolic dyfunction, MAFLD, Fatty liver

## Abstract

**Background:**

Metabolic Dysfunction-Associated Fatty Liver disease (MAFLD) among lean individuals is increasingly recognized. We aimed to compare the prevalence, metabolic characteristics, and outcomes of lean vs overweight/obese-MAFLD patients.

**Methods:**

Databases of Embase, Medline, and Web of Science were searched from inception till October 2023. Only cohorts adhering to the lean-MAFLD criteria as defined by the international consensus statement were included.

**Results:**

In the pooled analysis of 10,013,382 individuals, the prevalence of lean-MAFLD in the general population was 1.94% (95% CI 1.10–3.39%, *I*^2^ = 98.7%). Lean and overweight/obese-MAFLD patients had similar metabolic characteristics for blood pressure, LDL, TG, blood glucose, and HbA1c. There was an increased incidence rate and likelihood for liver-related mortality for lean-MAFLD vs overweight/obese-MAFLD [1.33 per 1000 patient-years (95% CI 1.28–1.39) vs 0.76 (95% CI 0.25–2.28), (OR 3.56 (95% CI 3.45–3.67), *p* < 0.01). There were similar incidence rates and odds ratios between lean vs overweight/obese-MAFLD for: (1) all-cause mortality [10.08 per 1000 patient-years (95% CI 9.93–10.23) vs 8.94 per 1000 patient-years (95% CI 4.08–19.57), (OR 1.92 (95% CI 0.01–220.57), *p* = 0.33)]; (2) cardiovascular-related mortality [2.53 per 1000 patient-years (95% CI 0.65–9.96) vs 2.07 per 1000 patient-years (95% CI 0.80–5.39), (OR 1.91 (95% CI 0.02–142.76), *p* = 0.58)]; and (3) cancer-related mortality [3.42 per 1000 patient-years (95% CI 3.33–3.51) vs 3.15 per 1000 patient-years (95% CI 1.21–8.19), (OR 1.99 (95% CI 0.29–13.52), *p* = 0.13).

**Conclusion:**

Lean-MAFLD patients have an equivalent metabolic burden compared to overweight/obese-MAFLD patients and thus a similar incidence rate of major extrahepatic complications. However, they have an increased risk of liver-related mortality.

**Graphical abstract:**

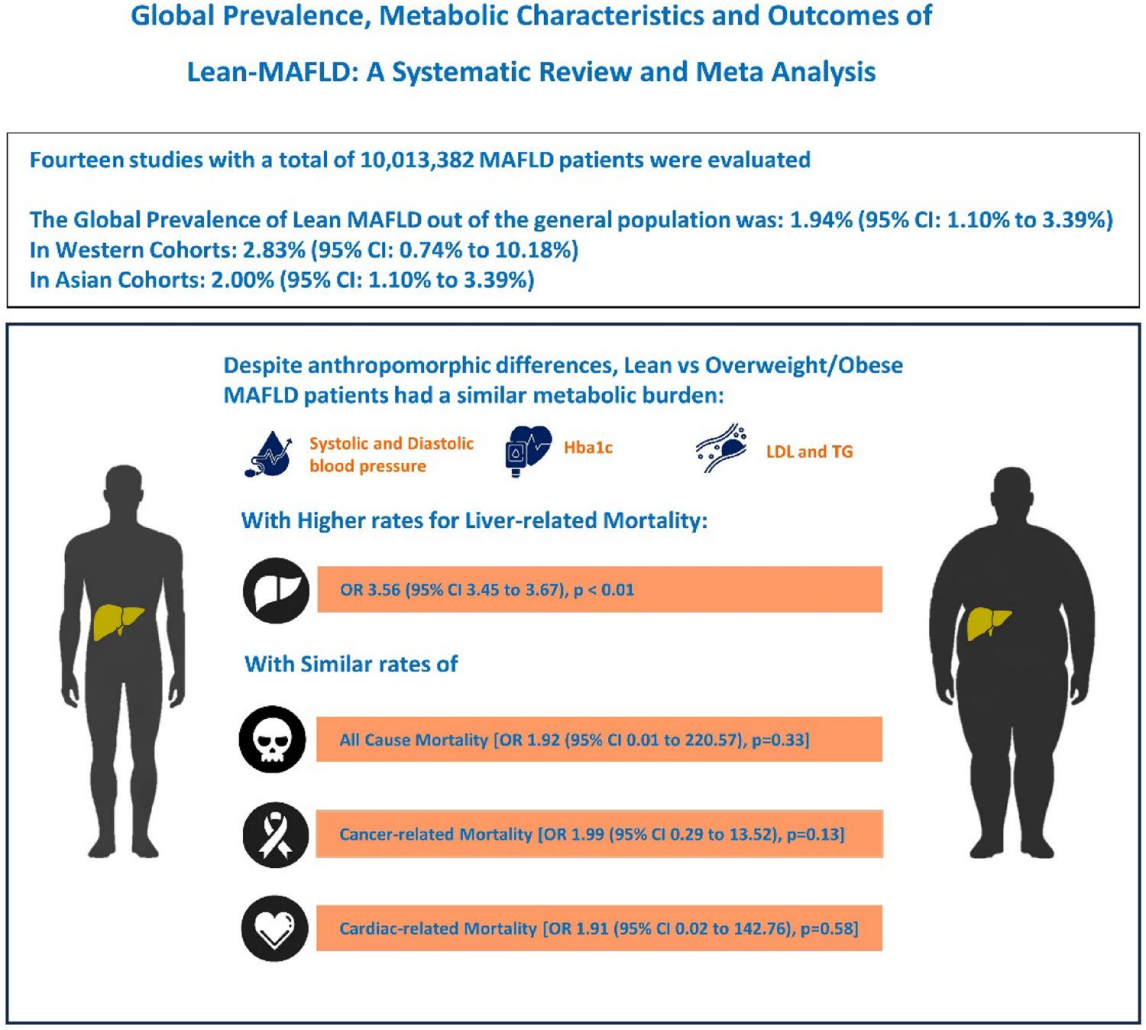

## Introduction

Fatty Liver Disease is a global health epidemic that is closely linked to excess adiposity, insulin resistance, and the metabolic syndrome. In 2020, an international consensus suggested a nomenclature change to metabolic (dysfunction)-associated fatty liver disease from non-alcoholic fatty liver disease (NAFLD) to better address the heterogeneity of clinical phenotypes and to emphasize the close link between fatty liver and metabolic dysfunction [1].

The MAFLD criteria classify patients in whom there is ≥ 5% hepatic steatosis into three non-exclusive phenotypes: (1) DM-MAFLD: patients with underlying type 2 diabetes; (2) Overweight/Obese-MAFLD: patients who have an ethnic-specific BMI of 25–29.9 kg/m^2^ (overweight) and ≥ 30.0 kg/m^2^ (obese) for individuals of Western descent, and 23.0–24.9 kg/m^2^ (overweight) and ≥ 25 kg/m^2^ (obese) for individuals of Asian descent; (3) lean-MAFLD: ethnic-specific BMI “normal weight” patients (BMI < 25 kg/m^2^ in Caucasians or < 23 kg/m^2^ in Asians) who satisfy two out of the following seven metabolic risk criteria: ethnic-specific increased waist circumference (≥ 102/88 cm in Caucasian men and women; ≥ 90/80 cm in Asian men and women), hypertension, hypertriglyceridemia, lower plasma HDL, prediabetes, elevated homoeostasis model assessment of insulin resistance score (HOMA-IR), or raised plasma high-sensitivity C-reactive protein (hsCRP).

Lean-MAFLD challenges the conventional paradigm of fatty liver disease which is typically associated with obesity. Lean patients, despite being of healthy, “normal” weight, were found to be metabolically unhealthy with a greater degree of visceral fat accumulation [2]. Moreover, they have been reported to be at greater risk of adverse liver and cardiovascular complications when compared with metabolically healthy lean individuals [2]. Similarly, patients defined as having normal-weight obesity have increased adiposity and visceral fat despite normal weight and have been shown to have an increased rate of metabolic risk factors and adverse cardiovascular outcomes [3]. This belies the imperfect nature of weight or BMI as measures of metabolic health and there is a need to better phenotype patients based on adiposity and metabolic burden.

In this systematic review and meta-analysis, we aim to describe the prevalence, metabolic characteristics and clinical outcomes of lean-MAFLD patients in comparison with overweight/obese-MAFLD patients. We hypothesize that using the metabolic (dysfunction)-first MAFLD criteria, lean-MAFLD patients will be of a similar metabolic burden and have comparable, if not worse clinical outcomes when compared with overweight/obese-MAFLD patients.

## Methods

### Search strategy and inclusion criteria

This systematic review and meta-analysis was conducted in accordance with PRISMA guidelines. Databases of Embase, Medline, and Web of Science were searched from inception until October 2023. Independent Data extraction was performed by Mark Cheah (MC) and Harry Crane (HC). Details of our search criteria are described in the appendix.

We included original research articles that identified Lean-MAFLD according to the MAFLD criteria [1] and applied ethnically appropriate cutoffs, especially for BMI and waist circumference. Written correspondence was sent to authors where there was lack of clarity if the lean-MAFLD criteria were applied correctly (appendix 3).

Data extraction was performed by MC and HC. Discordance and disagreements were resolved by mutual consensus.

### Quality assessment

Quality assessment was performed using the Joanna Briggs Institute (JBI) Critical Appraisal Tool for prevalence studies [4]. The tool appraises the quality of the cohort based on the appropriateness of the sample frame, sampling method, sample size adequacy, methods for identifying and describing the study condition, sufficient coverage, measure reliability, appropriate statistical analysis, and response rate.

### Statistical analysis

Statistical analyses were performed using R (4.3.1), The R Foundation for Statistical Computing, Vienna, Austria)) with RStudio (2023.0.1) using the meta, metaphor, and metasens packages. Geometric means were converted to approximate (raw) means and standard deviations (SD) using the “separate SD” method by Higgins et al [5] where appropriate. Studies with data presented in medians with interquartile ranges with or without minimum and maximum values were converted to means and SD using the formula by Wan et al [6].

To estimate the prevalence of lean-MAFLD, an analysis of pooled proportions was performed using a generalized linear mixed model (GLMM) with logit-link and Clopper–Pearson intervals. Clinical characteristics of lean-MAFLD were pooled using an analysis of single means using inverse variance weighting with the Restricted Maximum-Likelihood (REML) estimator.

For comparative analyses, pairwise comparisons for dichotomous variables were pooled using the Mantel–Haenszel method with the Paule–Mendel estimator and for continuous variables using inverse variance weighting with the REML estimator.

Knapp–Hartung adjustments were used to calculate the confidence intervals around the pooled effect. Random-effects models were used throughout given the expected heterogeneity of global data.

Publication bias was assessed using a Doi Plot with LFK index. The trim-and-fill method was applied to address publication bias where appropriate.

## Results

### Summary of included studies

**Fig. 1:**
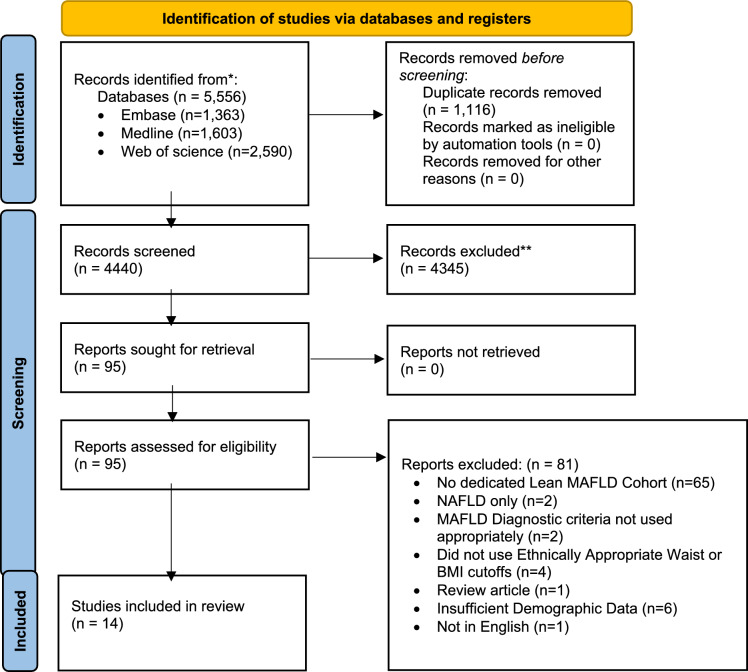
PRISMA flow diagram

A total of 5,556 articles were collated from Embase, Medline, and Web of Science. After removal of duplicates, a total of 4440 articles were screened with 95 articles assessed for full-text review (Fig. 1). A total of 14 studies were included in this meta-analysis (Table 1). This included one report from Australia, one report from Austria, two reports from China, one report from Hong Kong, two reports from Japan, one report from Mexico, four reports from South Korea, one report from Taiwan, and one from USA. There were nine health screening (healthy individuals), three prospective cohort, one population-based cross-sectional, and one comparative study of MAFLD patients vs a healthy cohort The average quality assessment of the collated studies using the JBI was 8.72, indicating that the studies included were of high quality.

**Table 1:** Summary of included studies

S. no	Author	Country	Study type	Cohort size	MAFLD (n, %)	Lean-MAFLD (n, %)	Diagnostic method	Quality
1	Bessho, 2022	Japan	Health screening	890	384 (43%)	63 (7.08%)	US	9/9
2	Cheng, 2021	Taiwan	Comparative study of MAFLD patients vs a healthy cohort	880	394 (44%)	65 (7.38%)	US	9/9
3	Cheng, 2022	China	Prospective cohort study	30633	6442 (21.0%)	531 (1.7%)	US	9/9
4	Dao, 2023	US	Prospective cohort study	13640	2619 (19.2%)	181 (1.32%)	US	9/9
5	Farrell, 2022	Australia	Prospective cohort study	4747	1756 (36.9%)	35 (0.74%)	FLI	9/9
6	Fukunga, 2022	Japan	Health screening	9100	2416 (26.5%)	275 (3.02%)	US	8/9
7	Goh, 2021	South Korea	Health screening	9935314	3561086 (35.8%)	193064 (1.94%)	FLI	9/9
8	Lee, 2023	Hong Kong	Population-based cross-sectional	1020	427 (41.8%)	30 (2.9%)	FS	9/9
9	Lim, 2023	South Korea	Health screening	42651	39614 (92.8%)	2631 (6.16%)	US	8/9
10	Ordoñez-Vázquez, 2022	Mexico	Health screening	3847	1536 (39.9%)	118 (3.06%)	FS	9/9
11	Park, 2021	South Korea	Health screening	6775	3021 (44.6%)	129 (1.9%)	US	9/9
12	Peng, 2023	China	Health screening	228	171 (75%)	31 (13.5%)	US	7/9
13	Semmler, 2021	Austria	Health screening	4178	2189 (52.4%)	221 (5.29%)	US	9/9
14	Sohn, 2022	South Korea	Health screening	2130	967 (45.4%)	47 (2.21%)	US	9/9

*FLI* Fatty Liver Index, *FS* Fibroscan®, *US* Ultrasound

The composition of the cohorts were: nine health screening (healthy individuals), three prospective cohort, one population-based cross-sectional, and one comparative study of MAFLD patients vs a healthy cohort. The majority of cohorts (*n* = 6) reported their hepatitis B/C rates compositely and this ranged between 1.8 and 6.7%. Three cohorts chose to exclude patients with viral hepatitis. For cohorts (*n* = 5) that did not report their viral hepatitis rates, the reported country-specific hepatitis B and C rates are in the range of 0.6–5.6% [7] and 0.4–0.7% [8] respectively. Overall, the composition of these cohorts and the overall low rates of viral hepatitis allows them to be reasonable representations of MAFLD patients as part of the larger general population for which they come from.

### Prevalence

In the pooled analysis of 10,013,382 individuals, the overall prevalence of MAFLD was 39.97% (95% CI 31.14% to 49.48%, *I*^2^ = 99.8%). The overall prevalence of lean-MAFLD in the general population was 1.94% (95% CI 1.10–3.39%, *I*^2^ = 98.7%), with an overall prevalence of lean-MAFLD among individuals with MAFLD of 5.40% (95% CI 3.54–8.15%, *I*^2^ = 98.4%).

Among Western Cohorts (Australia, Austria, Mexico, USA), the overall prevalence of MAFLD was 36.83% (95% CI 19.18% to 58.90%, *I*^2^ = 99.9%). The prevalence of lean-MAFLD in the general population was 2.83% (95% CI 0.74–10.18%, *I*^2^ = 98.8%), with an overall prevalence of lean-MAFLD among individuals with MAFLD of 8.03% (95% CI 2.32–2.43%, *I*^2^ = 97.6%).

Among Asian cohorts (China, Hong Kong, Japan, South Korea, Taiwan), the overall prevalence of MAFLD was 41.4% (95% CI 29.77–54.07%, *I*^2^ = 99.8%). The prevalence of of lean-MAFLD among Asian cohorts was 2.00% (95% CI 1.10–3.39%, *I*^2^ = 98.5%), with an overall prevalence of lean-MAFLD among individuals with MAFLD of 5.55% (95% CI 3.18–9.58%, *I*^2^ = 98.6%),

For non-obese-MAFLD (lean and overweight MAFLD), a pooled analysis of 19,948 indivduals from three studies was performed. The overall prevalence of non-obese-MAFLD in the general population was 15.61% (95% CI 3.73–46.92%) with the prevalence of of non-obese MAFLD among individuals with MAFLD being 43.85% (95% CI 18.35–73.07%).

### Metabolic characteristics

Lean-MAFLD patients expectedly had a lower BMI [mean difference (MD)-3.89 (95% CI – 6.83 to – 0.94), *p* = 0.01] and waist circumference [MD – 9.79 (95% CI – 11.45 to – 8.13], *p* < 0.01) when compared to overweight/obese-MAFLD patients. Despite this, lean-MAFLD patients had similar metabolic characteristics for systolic blood pressure, diastolic blood pressure, LDL, TG, blood glucose, and Hba1c, with only a clinically modest difference in HDL (MD 0.07 (95% CI 0.01–0.13), *p* = 0.02) when compared with overweight/obese patients (Table 2). Additionally, pooled odds ratios for hypertension (OR 1.17 (95% CI 0.67; 2.05), *p* = 0.31), diabetes mellitus (OR 1.19 (95% CI 0.44; 3.24), *p* = 0.67), ischemic heart disease (OR 1.85 (95% CI 0.37; 9.26), *p* = 0.24), and stroke (OR 0.65 (95% CI 0.01; 408.63), *p* = 0.55) were not signifcantly different between both groups (Table 3).

**Table 2:** Clinical, metabolic, and liver-related characteristics Lean vs Overweight/Obese-MAFLD

	Lean				Overweight and obese						
	Studies	Effect size	95% CI	*I*^2^(%)	Studies	Effect size	95% CI	*I*^2^(%)	MD	95% CI	*p* value
Age	11	55.54	53.17; 57.92	98.3	11	52.12	49.14; 55.10	99.8	3.28	2.09; 4.47	< 0.001
BMI	9	22.82	21.71; 23.95	99.9	11	26.53	25.09; 27.97	100.0	– 3.89	– 6.84; – 0.95	0.01
Waist Circumf	7	82.41	80.16; 84.65	98.6	7	92.38	89.36; 95.40	99.8	– 9.79	– 11.45; – 8.13	< 0.001
SBP	7	123.41	118.83;127.99	99.3	7	124.89	119.38; 130.40	99.3	– 0.73	– 3.01; 1.55	0.46
DBP	7	78.88	76.23; 81.53	95.3	7	80.16	76.20; 84.12	99.9	– 0.75	– 3.09; 1.59	0.46
Total Cholesterol	6	5.31	4.90; 5.71	94.8	5	5.08	4.79; 5.37	98.3	0.02	– 0.04; 0.09	0.42
LDL	6	3.23	3.03; 3.43	80.1	4	3.23	2.92; 3.55	99.4	– 0.04	– 0.09; 0.01	0.07
HDL	8	1.29	1.22; 1.37	98.5	7	1.21	1.14; 1.29	99.8	0.07	0.01; 0.13	0.02
Triglycerides	8	2.11	1.75; 2.47	99.6	7	1.89	1.78; 2.01	99.0	0.12	– 0.20; 0.45	0.39
Blood Glucose	8	6.01	5.92; 6.10	93.6	7	6.01	5.81; 6.20	99.8	0.06	– 0.02; 0.15	0.09
HbA1c	5	5.62	5.55; 5.69	58.7	5	5.64	5.46; 5.81	100.0	0.03	– 0.14; 0.20	0.66
Albumin	6	44.56	42.26; 46.86	99.5	5	43.94	42.04; 45.84	99.9	– 0.01	– 0.11; 0.11	0.96
ALT	9	30.83	26.57; 35.09	95.3	7	35.94	31.81; 40.07	97.6	– 7.17	– 11.29; – 3.05	0.005
AST	9	30.59	25.33; 35.8z	95.8	7	28.74	26.43; 31.06	98.4	0.34	– 5.05; 5.73	0.88
Platelets	5	262.65	221.27; 304.03	97.1	4	248.37	243.79; 252.95	96.8	4.43	1.29; 7.56	0.03
Creatinine	3	69.12	68.01; 70.23	0.0	2	77.19	75.20; 79.18	17.6	– 4.71	– 33.95; 24.55	0.29
FIB-4	4	1.24	1.07; 1.41	74.2	4	1.07	1.03; 1.09	67.3	0.17	– 0.06; 0.39	0.09
NFS	3	– 2.22	– 2.54; – 1.90	64.4	2	– 2.04	– 2.37; – 1.70	95.2	0.03	– 1.15; 1.21	0.81
LSM (MRE)	2	2.36	2.09; 2.62	71.1	2	2.42	2.38; 2.45	80.9	– 0.06	– 1.54; 1.42	0.67
CAP (FS)	3	281.69	266.03; 297.35	92.1	3	299.57	289.19; 309.95	96.5	– 16.21	– 27.32; – 5.10	0.02
LSM (FS)	3	4.39	4.04; 4.73	86.6	3	4.98	4.56; 5.39	95.6	– 0.55	– 0.75; – 0.36	< 0.01

*ALT* Alanine Transamine, *AST* Aspartate Transaminase, *BMI* Body Mass Index, *CAP* Controlled Attenuation Parameter, *DBP* Diastolic Blood Pressure, *FIB-4* Fibrosis-4 Index, *FS* Fibroscan®, *HDL* High-Density Lipoprotein, *LDL* Low-Density Lipoprotein, *LSM* Liver Stiffness Measurement, *MRE* Magnetic Resonance Elastography, *NFS* NAFLD Fibrosis Score, *SBP* Systolic Blood Pressure

**Table 3:** Pooled odds ratios, and metabolic and liver-related risk factors, Lean vs Overweight/Obese-MAFLD

	Studies combined	OR	95% CI	*I*^2^	*p* value
Hypertension	10	1.17	0.67; 2.05	96.9%	0.54
Diabetes	6	1.19	0.44; 3.24	95.5%	0.67
Dyslipidemia	7	1.66	0.73; 3.77	98.2%	0.18
Ischemic Heart Disease	3	1.85	0.37; 9.26	51.5%	0.24
Stroke	2	0.65	0.01; 408.63	6.9%	0.55
Significant Fibrosis	2	0.80	0.18; 3.4	0.0%	0.31
Advanced Fibrosis	2	1.68	0.03; 82.66	0.0%	0.34

For liver-related parameters, lean-MAFLD and overweight/obese-MAFLD patients had similar AST, Fibrosis-4 (FIB-4), NAFLD Fibrosis Score (NFS), Liver Stiffness Measurement (LSM) by Magnetic Resonance Elastography (MRE), but lower Controlled Attenuation Paramater (CAP) scores (MD – 16.21 (95% CI – 27.32 to – 5.10), *p* = 0.02), modestly lower LSM by Fibroscan® (MD – 0.55, 95% CI – 0.74 to 0.36), *p* < 0.01) and lower ALT (MD –7.17 (95% CI – 11.29 to – 3.04), *p* = 0.05) (Table 2). Pooled odds ratios for significant fibrosis (OR 0.80 (95% CI 0.18; 3.43), *p* = 0.31) and advanced fibrosis (OR 1.68 (0.03; 82.66), *p* = 0.33) were not significantly different between both groups (Table 3).

### Liver-related and clinical outcomes

For clinical outcomes, there was an increased incidence rate and likelihood for liver-related mortality for lean-MAFLD vs overweight/obese-MAFLD [1.33 per 1000 patient-years (95% CI 1.28–1.39) vs 0.76 (95% CI 0.25–2.28), (OR 3.56 (95% CI 3.45–3.67), *p* < 0.01). There were similar incidence rates and odds ratios between lean vs overweight/obese-MAFLD for: (1) all-cause mortality [10.08 per 1000 patient-years (95% CI 9.93–10.23) vs 8.94 per 1000 patient-years (95% CI 4.08–19.57), (OR 1.92 (95% CI 0.01–220.57), *p* = 0.33)]; (2) cardiovascular- related mortality [2.53 per 1000 patient-years (95% CI 0.65–9.96) vs 2.07 per 1000 patient-years (95% CI 0.80–5.39), (OR 1.91 (95% CI 0.02–142.76), *p* = 0.58)]; and (3) cancer-related mortality [3.42 per 1000 patient-years (95% CI 3.33–3.51) vs 3.15 per 1000 patient-years (95% CI 1.21–8.19), (OR 1.99 (95% CI 0.29–13.52), *p* = 0.13)] (Table 4).

**Table 4:** Clinical outcomes, Lean vs Overweight/Obese-MAFLD

	Studies	Participants	Events	Follow-up person Years	Incidence per 1000 person years (95% CI)	*I*^2^(%)	Studies	OR	95% CI	*I*^2^(%)	*p* value
All-cause Mortality											
Lean-MAFLD	3	193,320	17,439	1,729,258.23	10.08 (9.93; 10.23)	67.4	2	1.91	0.01; 220.59	88.4	0.33
Overweight/Obese-MAFLD	3	2,824,807	101,276	25,976,733.68	6.9 (4.24; 11.28)	98.2					
Cardiovascular-Related Mortality											
Lean-MAFLD	3	193,320	3195	1,729,258.23	2.53 (0.64; 9.96)	87.5	3	1.91	0.03; 142.76	78.2	0.58
Overweight/Obese-MAFLD	3	2,824,807	19,755	25,976,733.68	1.59 (0.80; 3.11)	96.3					
Cancer-Related Mortality											
Lean-MAFLD	2	193,320	5913	1,729,258.23	3.42 (3.33; 3.51)	0.0	2	1.99	0.29; 13.52	20.4	0.13
Overweight/Obese-MAFLD	2	2,824,807	41,782	25,976,733.68	1.61 (1.59; 1.62)	85.2					
Liver-Related Mortality											
Lean-MAFLD	2	193,320	2310	1,729,258.23	1.33 (1.28; 1.39)	74.1	2	3.56	3.45; 3.67	0.0	0.01
Overweight/Obese-MAFLD	2	2,824,807	9542	25,976,733.68	0.52 (0.23; 1.11)	87.6					

## Discussion

This is the first systematic review and meta-analysis examining the global prevalence, and metabolic and clinical outcomes of patients with lean-MAFLD in comparison with overweight/obese-MAFLD patients. As hypothesized, we found that lean-MAFLD patients have an equivalent metabolic burden when compared with overweight/obese-MAFLD and this likely accounts for their equal risk for the extrahepatic outcomes examined (cardiovascular, cancer-related, and all-cause mortality). On the other hand, lean-MAFLD is phenotypically distinct with liver disease onset at a lower set-point BMI-driven by disparate and unique mechanisms compared to overweight/obese-MAFLD. This contributes toward their increased liver-related mortality compared to overweight/obese-MAFLD. Our study answers previous controversey among lean NAFLD cohorts that report higher liver-related mortality despite having a better metabolic profile [9]. In addition, it highlights the strengths of the MAFLD criteria that is able to positively and correctly identify lean/normal individuals who are metabolically unhealthy with a high risk of adverse extrahepatic and hepatic outcomes.

A key contributor to hepatic inflammation in lean-MAFLD is a dysregulation of metabolic adaptation. Early in disease, bile acids levels are consistently increased in lean when compared with overweight/obese-MAFLD patients and dampens liver inflammation. However, over time, endotoxemia contributed by differences in gut microbial dysbiosis among lean-MAFLD but not healthy individuals or overweight/obese-MAFLD patients results in alterations in the epigenomic and transcriptome including upregulation of Toll-Like Receptor 4 (TLR4). This contributes to a failure of bile acid mediated anti-inflammatory signaling resulting in increased cytokine activation driving liver inflammation and long-term adverse hepatic sequelae [10]. In addition, telomere attrition mediated at least in part by increased reactive oxygen species generation is more pronounced among lean-MAFLD cohorts when compared with overweight/obese patients and might contribute toward increased mortality [11].

Another contributory factor to the increased liver-related mortality is the greater degree of sarcopenia found among MAFLD patients who are lean [12]. This may be exacerbated to a greater extent with the onset of cirrhosis, which in turn is associated with an increased risk of adverse liver-related outcomes [13]. This is a key issue as weight loss is the typical initial recommendation for the management of MAFLD and careful consideration needs to be given for patients who are lean with MAFLD. Our findings are particularly relevant in the current era of potent pharmacological weight loss drugs where there are growing concerns for worsening sarcopenia associated with rapid weight loss. This was recently shown using data from the dual x-ray absorptiometry (DEXA) subgroup of the STEP-1 trial evaluating Semaglutide for weight loss. That sudy reported that although there was a 8.36 kg fat mass loss in the semaglutide group, there was an accompanying 5.26 kg loss of lean muscle mass (comprising ~ 40% of total weight loss) [14]. In the SURMOUNT-1 trial evaluating Tirzeptide for weight loss, the investigators reported a -33.9% fat mass loss but a worrying 10.9% lean mass loss [15]. While mouse models purport that GLP1-receptor agonists (GLP1-RA) ameliorate sarcopenia and muscle atrophy [16], this is not reflected in human data. Similar effects of weight loss-associated lean muscle loss have been reported in a recent meta-analysis of bariatric surgery patients. That study reported a greater than 8 kg of lean body mass loss during the first year post bariatric surgery [17]. The findings suggest that lean muscle loss is associated with the rapidity of weight loss rather than a drug-class specific effect. Thus, perhaps, the positive pleiotropic effects of GLP1-receptor agonists need to be balanced against the risk of sacropenia and the need to achieve more gradual weight loss targets in lean-MAFLD patients, particularly those with more advanced fibrosis.

An unresolved issue remains that there are no current, nor in-development treatments that specifically benefit patients with lean-MAFLD. The ENLIVEN (Pegozafermin, fibroblast growth factor 21 (FGF21) analog) [18] and SEMA-NASH (Semeglutide) [19] trials outrightly exclude patients with BMI < 25 kg/m^2^, while other trials, such as MASTERO NASH (Resmetirom, thyroid hormone receptor-β agonist) [20], HARMONY (Efruxifermin, FGF21 analog) [21], FALCON (Pegbelfermin, FGF21 analog) [22], and FLINT (Obeticholic Acid, Farsenoid X nuclear receptor ligand) [23], have an average BMI > 35 kg/m^2^ across treatment and control groups, suggesting predominantly overweight/obese participants. The underrepresentation of lean-MAFLD patients in these trials limits the generalizability of the therapeutic agents and sets a pressing need for trials inclusive of patients with lean-MAFLD. Practically, patients with lean-MAFLD will require therapies that target visceral and hepatic fat loss, inflammation, and fibrosis, while preserving lean muscle mass and improving liver-related and metabolic outcomes. This might require a more nuanced approach to treatment with dose reduction of weight loss agents such as GLP1-RA (where needed), possibly in combination with agents that also improve lipid and glycemic profiles but having minimal effects on weight such as resmetirom [20] and FGF21 analogues [22].

We found that the global prevalence of lean-MAFLD (1.94%) was similar among Western (2.83%) and Asian (2.00%) cohorts. This is half the estimated global prevalence of lean NAFLD (5.1%) from another meta-analysis [24]. The difference is likely attributed to the more stringent lean-MAFLD criteria identifying the group of lean patients having a greater metabolic burden. In a study using the NHANES III cohort (1999–2015), patients who were MAFLD ( – ) NAFLD ( +) (hence were lean NAFLD patients who did not have diabetes and who had less than 2 metabolic risk factors) were shown to have a lower metabolic burden, lower BMI (21.8 ± 0.2), lower waist circumference, lower proportions of significant and advanced hepatic fibrosis, with lower rates of all-cause, cardiovascular, cancer, and other cause mortatily when compared with patients who were MAFLD ( +) NAFLD ( +) and patients who were MAFLD ( +) NAFLD ( – ). This indicates that lean NAFLD patients who did not satisfy the MAFLD criteria have significantly more benign outcomes [25]. In another NHANES cohort (1999–2018) which compared lean patients with or without NAFLD and/or MAFLD, lean patients who were MAFLD ( +) NAFLD ( – ) and MAFLD ( +) NAFLD ( +) were shown to have higher all-cause and cardiovascular mortality when compared with lean-MAFLD ( – ) NAFLD ( +) patients [26]. Another recent meta-analysis also demonstrated that the metabolic burden of lean NAFLD patients is lower than that of overweight/obese NAFLD patients [27]. These findings imply a dilutional effect of metabolic risk factors in studies reporting on lean NAFLD, where patients with the more benign MAFLD ( – ) NAFLD ( +) are mixed with lean-MAFLD ( +) patients. In sum, by not applying the MAFLD criteria, lean fatty liver patients who have a higher metabolic burden might potentially be masked and underreported in lean NAFLD cohorts; conversely, the lean-MAFLD criteria identifies the proportion of lean fatty liver patients who are at risk of worse clinical outcomes and require more pro-active management.

In real-world practice, MAFLD is not a static disease and patients may accrue metabolic risk and potentially have changes in phenotype either in a positive or negative direction. This was examined in a longitudinal observational study by Younes et al [28], where liver biopsy proven lean caucasian NAFLD patients from a multicentre cohort were followed for a median of 94 months. In that study, the majority of lean patients remained lean (77.5%) and yet developed incident diabetes and had complications including cardiovascular events, extrahepatic malignancy, and liver-related events and HCC similar to their overweight and obese patients. This was not explained by concomitant weight gain nor PNLP3A genotype. Importantly, the development of diabetes was an independent predictor of cardiovascular events and extrahepatic malignancy, indicating the importance of metabolic risk factors in the prognosis of patients with MAFLD.

Another issue is the impact of the metabolic dysfunction-associated steatotic liver disease (MASLD) criteria is the MAFLD criteria among lean patients. The diagnosis of MASLD is established when a patient meets any one of the five cardiometabolic criteria [29]. This approach aims to emphasize the role of metabolic dysfunction while ensuring that the majority of individuals with NAFLD are reclassified under MASLD. However, the key limitation of the MASLD criteria, especially toward lean patients, is the equal weighting of a single metabolic criterion for diagnosis which does not account for differences in clinical outcomes or treatment. For instance, lean individuals with diabetes—a recognized driver of disease progression—are considered equivalent to those lean individuals with isolated hypertension. This lack of differentiation has several implications. First, from an epidemiological point of view, patients with a higher metabolic burden and higher risk of adverse outcomes may become underrepresented when grouped under a single umbrella of MASLD. Further, it would be impossible to effectively report individual phenotypes (e.g., MASLD: lean with hypertension), especially when patients may have more than one metabolic risk factor. Finally, this suboptimal classification will have an impact on the design of future clinical trials for lean patients as the inclusion of individuals with varying metabolic burdens may obscure differences in treatment efficacy, potentially leading to inadequate reporting of treatment outcomes. The lean-MAFLD criteria, on the hand, identify a proportion of lean patients with a higher metabolic burden requiring urgent clinical attention. In our manuscript, we demonstrated that lean-MAFLD patients had similar clinical outcomes for all-cause, cancer-related, and cardiac-related mortality when compared with overweight/obese-MAFLD patients, with higher liver-related mortality among lean-MAFLD patients. We believe that the lean-MAFLD better risk stratifies lean patients based on their higher metabolic burden.

Our study has several limitations. First, the Doi plot and LKF index (5.51) suggest publication bias when evaluating the global prevalence of lean-MAFLD (appendix). During our systematic review, we found that the majority of the MAFLD cohorts screened and excluded from the study did not sub-classify lean-MAFLD as a separate subgroup. In addition, we found that there were six studies that did not use ethnic appropriate cutoffs (of waist circumference and BMI) in defining lean-MAFLD and were thus also excluded. To compensate for this, we used the trim-and-fill method to approximate the true prevalence of lean-MAFLD. Second, as expected with global data, there was high heterogeneity detected in the majority of our analyses. We recognize that heterogeneity is often viewed with concern, we would like to emphasize that it does not diminish the utility of the analysis itself. In principle, we believe that the MAFLD criteria of having at least two metabolic risk factors in patients with lean-MAFLD inherently sub-select a group of lean patients of a higher metabolic burden. While heterogeneity may indicate a greater degree of dispersion around the true effect size, we believe that the effect sizes from our analysis accurately portray the higher metabolic burden associated with lean-MAFLD and are comparable to patients with overweight/obese-MAFLD. Further, heterogeneity is anticipated in global epidemiological studies and has been well reported [30]. This is likely to be contributed by multiethnic societies and reflects differences in genetic, dietary, and environmental factors unique to each country, even when geographically close. Statistically, we have sought to compensate for the heterogeneity encountered by applying random-effects models throughout our analysis. Third, there were insufficient data to compare metabolic and outcome data of lean NAFLD, and healthy lean patients. Finally, we lacked outcome data on incident development of metabolic risk factors such as new onset diabetes or dyslipidemia which may add insight into the prognostic value of the MAFLD criteria.

In summary, our findings highlight the ultility of the MAFLD criteria, which is able to correctly classify patients that share an equivalent metabolic burden and a similar risk of extrahepatic adverse outcomes, regardless of anthropomorphic differences between lean and overweight/obese patients. We report increased liver-related mortality among lean-MAFLD patients which is likely contributed by dysregulation of metabolic adaptation, telomere shortening, and sarcopenia. Nonetheless, there remains challenges in raising awareness in correctly identifying metabolically unhealthy lean-MAFLD patients. Finally, there is an urgent need for directed and specific clinical trials for lean-MAFLD patients that target fat loss and inflammation explicity in the liver, while preserving lean muscle mass and improving both metabolic and liver-related outcomes.

## Data Availability

Studies included in the meta analysis are collated in Table 1.

## Appendix 3

Authors where written correspondence was sent

1. Yuan QL, Wang H, Gao P, et al. Prevalence and Risk Factors of Metabolic-Associated Fatty Liver Disease among 73,566 Individuals in Beijing, China. *International Journal of Environmental Research and Public Health.* 2022;19(4)
2. Cheng YM, Kao JH, Wang CC. The metabolic profiles and body composition of lean metabolic associated fatty liver disease. *Hepatol Int.* 2021;15(2):405–412.
3. Li H, Guo M, An Z, et al. Prevalence and Risk Factors of Metabolic Associated Fatty Liver Disease in Xinxiang, China. *Int J Environ Res Public Health.* 2020;17(6).
